# Limitations of Mechanistic Model of Explanation in Biological Psychiatry

**DOI:** 10.1192/j.eurpsy.2022.1724

**Published:** 2022-09-01

**Authors:** M. Salcedo Gómez, C. García Aguilar

**Affiliations:** 1 National Autonomous University of Mexico, Biotecnology, Mexico, Mexico; 2 National Autonomous University of Mexico, Institute Of Philosophical Research, Mexico, Mexico

**Keywords:** biological psychiatry, mechanistic model of explanation, RDoC, multiple realization

## Abstract

**Introduction:**

The National Institute for Mental Illness (NIMH) launched in 2008 a project based on ‘precision medicine,’ called Research Domain Criteria (RDoC). This precision medicine approach, novel in the context of psychiatry, proposes to identify the “fundamental components of behavior,” determining their range of variations from normality to abnormality and identifying their instantiations at different levels of the biological mechanism. To achieve its goal, an essential task of the RDoC initiative has been to identify and classify psychological constructs associated with psychopathology and to cut them off at a finer degree of granularity, presumably in order to have a greater chance of finding the biological mechanisms which implement every resultant part.

**Objectives:**

Our work aims to show the limitations that psychiatry faces when assuming the mechanistic model of explanations. We will show how, if we accept the phenomenon of multiple realization, it is not plausible to expect that the RDoC initiative will be successful in their enterprise to track single or precise causal mechanisms for every construct identified at the cognitive level.

**Methods:**

Philosophical argumentation

**Results:**

No results.

**Conclusions:**

We conclude that an approach that aims to identify single functional units and to dig down at a “fundamental level” to find their neural or genetic implementation should not only be reconsidered in terms of the phenomenon of multiple realization, but also leaves a gap in our understanding of the complex structures that are found at the cognitive-functional level and whose dysfunctions would be of great explanatory relevance concerning mental disorders.

**Disclosure:**

No significant relationships.

